# Late rectal toxicity determined by dose–volume parameters in computed tomography‐based brachytherapy for locally advanced cervical cancer

**DOI:** 10.1002/cam4.603

**Published:** 2016-01-24

**Authors:** Yong‐Chun Zhou, Li‐Na Zhao, Ning Wang, Jing Hu, Xiao‐Huan Sun, Ying Zhang, Jian‐Ping Li, Wei‐Wei Li, Jun‐Yue Liu, Li‐Chun Wei, Mei Shi

**Affiliations:** ^1^ Department of Radiation OncologyXijing HospitalFourth Military Medical UniversityXi'an710032China

**Keywords:** Computed tomography‐based brachytherapy, dose‐volume histogram parameters, late side effects, locally advanced cervical cancer, rectum

## Abstract

The aim of this study was to observe the relationship between dose–volume histogram (DVH) parameters and rectal late side effects (LSE) in computed tomography (CT)‐based brachytherapy (BT) for patients with locally advanced cervical cancer. In total, 144 cervical cancer patients received external beam radiotherapy and CT‐based BT. The data from 111 survival cases with pelvic local control (LC) were used to analyze the relationship between DVH parameters and rectal LSE. The total doses, manifesting 2, 1, and 0.1 cm^3^ (D_2cc_, D_1cc_, and D_0.1cc_) of the rectum, and D_90_ for high‐risk clinical target volume (HR CTV) were computed and normalized to 2 Gy fractions (EQD2) using a linear‐quadratic model. The rectal LSE were evaluated by the late effects in normal tissues‐subjective, objective, management, and analytic (LENT‐SOMA) scale. A dose–response relationship was evaluated by probit analyses. For all patients, the total rate of rectal LSE was 56%, and the rate of ≥Grade 2 LSE was 27.4%. For the 111 survival cases with pelvic LC, the total mean for D_2cc_ was 71.23 ± 5.54 Gy for the rectum, and the D_2cc_, D_1cc_, and D_0.1cc_ values for Grades 2 and 3 were higher than those for Grades 0 and 1. In addition, the number of complications increased, and the complications became more severe as the dose increased, with a dose of 73.5 Gy resulting in a 10% probability of ≥Grade 3 LSE. In conclusion, DVH parameters could predict the incidence and grades of rectal LSE in CT‐based BT. D_2cc_ showed an excellent predictive value, and 73.5 Gy for D_2cc_ of the rectum might be considered as an alternative dose limit.

## Introduction

A combination of external beam radiotherapy (EBRT), concurrent cisplatin‐based chemotherapy and brachytherapy (BT) is the standard treatment for locally advanced cervical cancer patients. The GYN GEC‐ESTRO working group has published recommendations concerning three‐dimensional (3D) image‐based BT with either computed tomography (CT) or magnetic resonance imaging (MRI) in cervical cancer 1, 2, 3, 4. Due to its relatively accurate delineation of anatomical structures and ease of implementation, 3D CT‐based BT has received more attention and has been routinely used in some institutions worldwide in recent years 5. Some reports have shown favorable local control (LC) rates (>90%) and a low incidence of late side effects (LSE) of organs at risk (OAR) by CT‐based BT 6.

Compared with point 2D parameters, such as point A and point B, dose–volume histogram (DVH) parameters with the effective visualization of target volumes and OAR were considered to be more accurate for dose assessment in 3D BT 7. Furthermore, some DVH parameters, such as D_90_‐HR CTV (the dose covering 90% of the high‐risk clinical target volume) for tumors and D_2cc_ (the minimum dose in the 2cc most irradiated tissue volume) for OAR, have been demonstrated to be predictive of the tumor control probability and occurrence of LSE for OAR 1, 2, 8. Recently, a few studies indicated that DVH parameters, such as D_5cc_ or D_2cc_ might be more reliable for prediction of the risk of ≥Grade 2 late rectal complications by CT‐based BT 9, 10.

Currently, 3D BT is not been widely used throughout the world, and even in some developed countries, the rate is only approximately 50% 11. Although some consensus regarding 3D BT has been achieved, the setting of the standard was mainly based on MRI, and the data concerning CT‐based BT, which represented the majority of 3D BT, was relatively limited. Furthermore, few data were reported regarding the relationship between DVH parameters and the effects on OAR in CT‐based BT, especially for the evaluation of rectal toxicity, which needs to be further elucidated.

In this study, we report the clinical outcomes of 144 locally advanced cervical cancer patients treated with CT‐based BT in China and further analyzed the relationship between the DVH parameters and LSE of the rectum in 111 survival cases with pelvic LC. The patients with local failure were excluded from this study because of the similar symptoms between local failure and rectum toxicity. This study, to the best of our knowledge, is the first providing detailed data for evaluating the role of DVH parameters in the prediction of rectal LSE in the setting of CT‐based BT for locally advanced cervical cancer in China.

## Materials and Methods

### Patients and treatment

Between July 2008 and December 2009, 144 consecutive patients (FIGO stage IB2‐IIIB, according to the pelvic examination) received radical radiotherapy in the Department of Radiation Oncology, Xijing Hospital, Fourth Military Medical University, China. All the patients were treated with a combination of EBRT and CT‐based BT with or without concurrent cisplatin (40 mg/m^2^ per week). The EBRT was performed using 3D conformal technology, the entire pelvic irradiation dose was 40–50 Gy in 20–25 fractions, and some cases followed with central shielding by 8–20 Gy in 4–10 fractions. Thus, the total dose of EBRT was 50–60 Gy. Then, intracavitary BT or combined intracavitary/interstitial BT was performed using a CT‐based procedure with 4–7 fractions of 6 or 7 Gy (the dose range from 24 Gy/4F to 42 Gy/6F) twice a week. The HR CTV dose was prescribed for planning, all doses were converted into the equivalent dose in 2 Gy fraction (EQD2), the EQD2 of D90 for HR CTV ranged from 62.72 to 105.91 Gy.

As a routine examination, MRI was used to assess the range and the change of tumors at diagnosis and before BT, respectively, which was also necessary to guide the delineation of the target volumes.

During the follow‐up stage, clinical examinations, MRI‐based imaging tests, and LSE scores were performed every 3 months for the first year and twice annually thereafter.

### Target contouring and treatment planning

Before CT‐based BT, proper preparation of the bowel and bladder is necessary. On the day before BT, a soaking solution with folium sennae was ingested to achieve an empty sigmoid and rectum. On the treatment day, 120 mL saline was instilled into the empty bladder before the CT scan and BT implementation. The pelvic transverse images were acquired by CT simulator (Philips Medical Systems, Cleveland, USA) and transferred to a treatment planning system (Nucletron Systems, Veenendaal, The Netherlands). The slice thickness of CT scan was 5 mm, the upper border was parallel to renal hilus and the lower border was the level of 3 cm below ischial tuberosity.

Based on CT‐standardized Contour Guidelines^12^, referred MRI image and GYN GEC ESTRO recommendations 1, 2, HR CTV and OAR (rectum, sigmoid, and bladder) were contoured on CT image. The plan for CT‐based BT was calculated by treatment planning system and then implemented using an afterloading system (Nucletron).

### DVH analysis

In order to eliminate the interfering factors from death after a short time of radiotherapy and local residue or recurrence, which may miss the occurrences of rectal LSE or have similar symptoms of rectum, such as tenesmus, frequent stool, pain, and bleeding, the survival patients with pelvic LC were used to analyze the relationship between the DVH parameters and rectal LSE. The patient characteristics and treatment factors are shown in Table 1.

**Table 1:** Characteristics of 111 patients and their treatment

Clinical variable	Numbers for grade 0–1	Numbers for grade 2–3	*P* values
Age (Median age ranging)	52 (27–74)		
<55	48	20	0.27
≥55	26	17	
FIGO stage (Ib2‐IIIb)			
Ib2‐II	13	2	0.077
III	61	35	
Histological diagnosis			
Squamous cell carcinoma	111		
Maximum tumor dimension (cm)			
≤5	51	24	0.667
>5	23	13	
Weekly cisplatin			
No	8	6	0.356
Yes	66	29	
Brachytherapy			
Methods			
Intracavitary	57	31	0.408
Combined intracavitary/interstitial	17	6	
Fraction			
4–5	34	15	0.672
6–7	40	22	
Overall treatment time (days)			
<60	38	17	0.688
60–79	36	20	
Treatment results			
No distant metastasis	70	33	0.437
Distant metastasis	4	4	

*P* value for rectal LSE (Grade 0–1 vs. Grade 2–3).

The DVHs for each patient were generated for each BT fraction, and the parameters were described by D_90_ for HR CTV and by D_2cc_, D_1cc_, D_0.1cc_ for the rectum. All total doses were converted to EQD2 using a linear‐quadratic model with *α*/*β* ratios of 10 Gy for HR CTV and 3 Gy for the rectum.

### LSE scoring

The rectal LSE were evaluated by the Late Effects in Normal Tissues‐Subjective, Objective, Management and Analytic (LENT‐SOMA) scale 13, 14, 15. In accordance with some reports 16, the highest score in any one term was recorded for the final grade of toxicity in each patient. The minimum interval from the end of radiotherapy to evaluation was 6 months.

### Statistical analysis

The actuarial overall survival (OS), pelvic LC, progression‐free survival (PFS), and rectal LSE rates were calculated using the Kaplan–Meier method. The measurement data, including DVH parameters, were compared by analysis of variance (ANOVA), and the count data, such as the rate of LSE, were compared by the chi‐square test. In addition, the dose–response relationship was evaluated by probit analyses, and the dose–response curves were created (logit model). All statistical analyses were performed using SPSS 18.0 (SPSS, Chicago, IL, USA).

## Results

### Treatment outcomes

**Figure 1:**
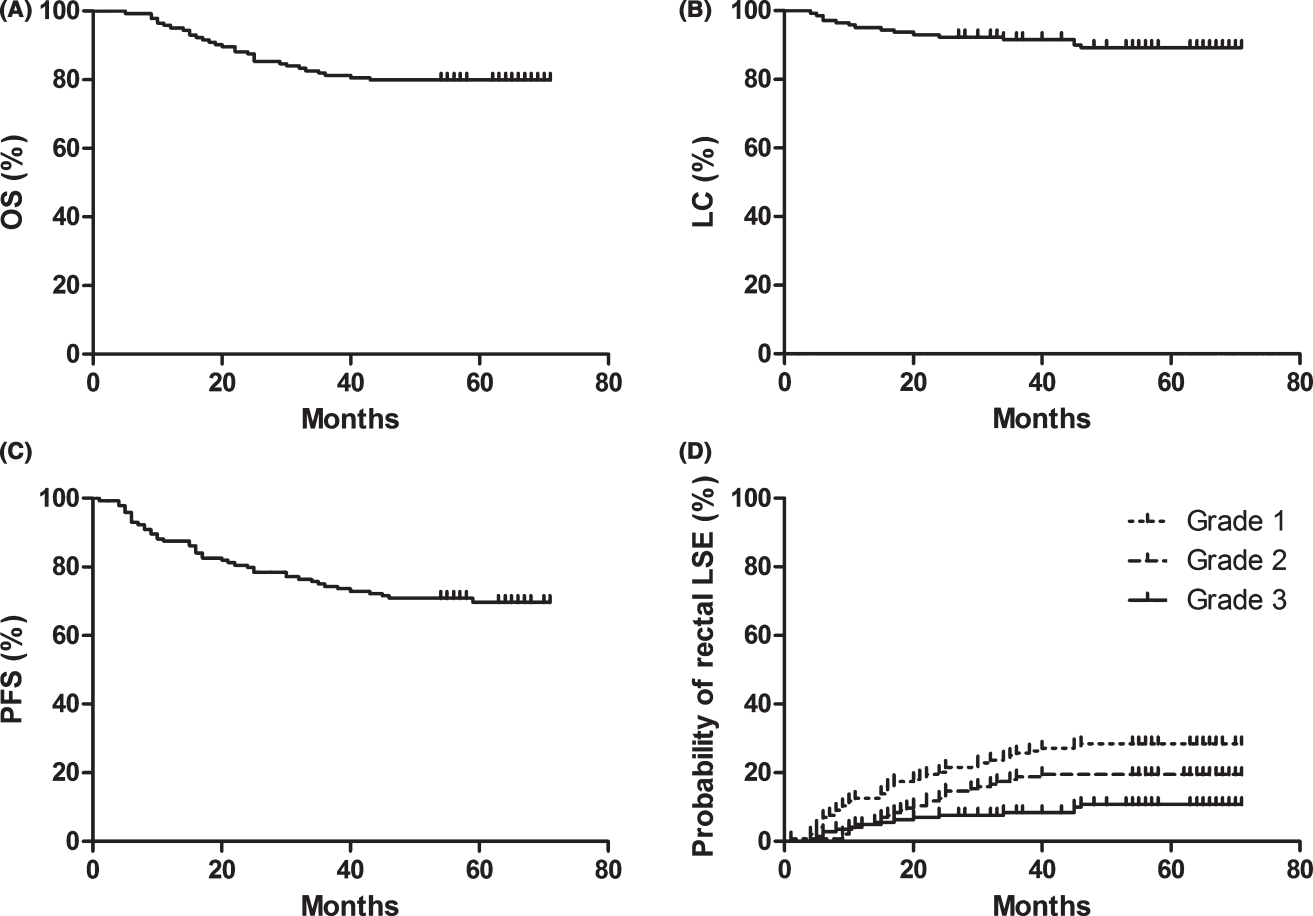
The overall survival (OS) (A), local control (LC) (B), progression‐free survival (PFS) (C) and the accumulated rates of rectal late side effects (LSE) by Grade (D) for all of 144 patients.

The median follow‐up was 58 months (5 to 71 months). The 5‐year OS, pelvic LC, and PFS for all the patients were 79.9, 89.3, and 69.7%, respectively (Fig. 1A–C).

### Occurrence of rectal LSE

The actuarial rate of rectal LSE was 56%. Thirty‐seven (28.5%) patients were classified as Grade 1, 24 (17.0%) as Grade 2, and 15 (10.4%) as Grade 3 (Fig. 1D). Among these patients, the symptoms associated with Grade 2 rectal LSE were occasional bleeding or intermittent diarrhea caused by tenesmus. For Grade 3 LSE, 12 patients developed slight daily bleeding, and the remaining three patients suffered from persistent bleeding and required red blood cell transfusions.

### Patient and treatment characteristics were unrelated to rectal LSE

In total, 111 survival cases with pelvic LC were followed‐up for at least 48 months, and the data were used to analyze the relationship between the DVH parameters and rectal LSE. The characteristics of these patients, such as patient age, FIGO stage, tumor diameter, weekly concurrent cisplatin administration, overall treatment time, BT methods, and fraction schedules showed no significant relationship with occurrence of rectal LSE (Table 1).

### The relationship between DVH parameters and rectal LSE

The values of all the DVH parameters for patients with all grades of rectal LSE are summarized in Table 2. The mean (±SD) D_90_ for HR CTV, D_2cc_, D_1cc_, and D_0.1cc_ values for the rectum were 86.69 ± 8.91, 71.23 ± 5.54 Gy, 75.25 ± 6.29 Gy, and 84.48 ± 8.02 Gy, respectively, and the values of D_2cc_, D_1cc_, and D_0.1cc_ were significantly higher in patients with Grade 2 and 3 rectal toxicity than in those with Grades 0 and 1 (*P *< 0.05). However, no significant difference in D_90_‐HR CTV was observed among all grades of rectal LSE. Furthermore, all DVH parameters between Grades 0 and 1 rectal LSE showed no significant difference.

**Table 2:** Dose levels for LSE of rectum

Grade for LSE	Numbers	DVH parameters (Gy)			
		D90‐HR CTV	D_2cc_	D_1cc_	D_0.1cc_
Grade 0	42	85.96 ± 8.55	69.94 ± 5.74	73.68 ± 6.41	82.32 ± 7.63
Grade 1	32	85.56 ± 10.49	68.97 ± 4.87	72.92 ± 5.77	82.09 ± 8.02
Grade 2	22	88.02 ± 8.21	72.95 ± 4.31^a^	77.19 ± 5.01^a^	87.08 ± 7.07^a^
Grade 3	15	88.98 ± 6.45	77.11 ± 3.96^a^^,^ b	81.80 ± 4.87^a^^,^ b	91.82 ± 5.21^a^^,^ b

LSE, late side effects; DVH, dose–volume histogram; HR CTV, high‐risk clinical target volume.

a *P *< 0.05 (vs. Grade 0 and 1).

b *P *< 0.05 (vs. Grade 2).

For the dose stratification analysis, the patients were classified into four groups according to the D_2cc_ values of the rectum by intervals of 5 Gy ranging from 65 to 80 Gy. With increasing D_2cc_ values, especially when the cutoff value was greater than 70 Gy, the rates and grades of rectal LSE increased remarkably (*P *< 0.05). The actuarial rates of rectal LSE for the patients are shown in Table 3.

**Table 3:** Dose distribution for LSE of rectum

Grade for LSE	Numbers (%) in different D_2cc_ (Gy) ranges			
	<65	65–70 (<70)	70–75 (<75)	75–80 (<80)
Grade 0	10 (52.63)	10 (38.46)	12 (35.29)	10 (31.25)
Grade 1	9 (47.37)	10 (38.46)	9 (26.47)	4 (12.50)
Grade 2	0 (0)	6 (23.08)	9 (26.47)	7 (21.88)
Grade 3	0 (0)	0 (0)	4 (11.77)	11 (34.37)
Grade 0–1	19 (100)	20 (76.92)	21 (61.76)	14 (43.75)
Grade 2–3	0 (0)	6 (23.08)^a^	13 (38.24)^a^^,^ b	18 (56.25)^b^

LSE, late side effects.

a *P *< 0.000 in D_2cc_ cutoff of 70 Gy.

b *P *=* *0.008 in D_2cc_ cutoff of 75 Gy.

**Figure 2:**
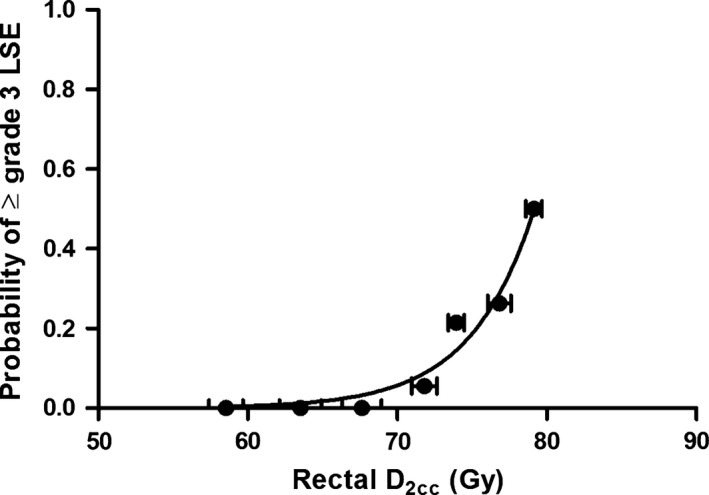
Relationship between D_2cc_ and severe rectal LSE (late side effects). For illustration of data points, patients have been pooled into dose intervals of 5 Gy for D_2cc_ ≤ 70 Gy and 2.5 Gy for D_2cc_ between 70 and 80 Gy.

In further dose–response analyses, a significant dose effect was found for D_2cc_ of the rectum and complications ≥Grade 3 (Table 4). The probit curves (Fig. 2) showed the dose effect for ≥Grade 3 rectal LSE, in which a dose of 73.5 Gy results in a 10% probability of ≥Grade 3 LSE.

**Table 4:** Probability of G3–G4 rectal LSE according to D_2cc_

Dose volume	Probability of EQD2 for ≥Grade 3 rectal LSE (Gy) for the incidence rates shown (95% CI)			
	5%	10%	20%	*P* value
D_2cc_	72.0 (55.9–75.4)	73.5 (60.9–76.6)	75.4 (66.8–78.1)	0.005

LSE, late side effects; EQD2, equivalent dose in 2 Gy fraction; CI, confidence interval.

## Discussion

Combined with EBRT and concurrent chemotherapy, BT plays an important role in the treatment of locally advanced cervical cancer. However, the clinical outcome using conventional 2D planning BT is not very satisfactory. Some previous retrospective studies and a recent phase II study showed a relatively poor LC rate (67–86%), especially in patients with late‐stage cervical cancer (stage III or IV by the FIGO staging system). Moreover, the rate of late toxicities was high, especially for Grade 3 and 4 toxicities because the dose of point A was the reference dose for 2D planning 17. Compared with 2D BT, satisfactory clinical outcomes were observed in 3D CT‐based BT, which could enable the delivery of a very high dose to the tumor while avoiding the OARs. Thus, 3D CT‐based BT could improve the LC rate while lowering the LSE rate 6, 18. Recently, as the gold standard method for target delineation, MR‐based BT has shown promising outcomes 19, 20. After the recommendation of GYN GEC‐ESTRO in the 3D IGBT of locally advanced cervical cancer was published, we practiced this guideline in our clinical work and also achieved favorable curative effects, particularly with respect to OS, LC, PFS, and severe rectal LSE.

For the BT of locally advanced cervical cancer, a conventional 2D plan, which untruly reflect the anatomical structures, often overestimates the tumor dose and underestimates the OAR doses. In relative terms, a 3D CT‐based plan can provide more accurate information regarding target volume coverage and an appropriate evaluation of OARs^21^, ^22^, ^23^, ^24^, ^25^. According to the ESTRO project for 3D image‐based BT, the point doses in a 2D plan were replaced, and the prescribed dose was evaluated by DVH parameters, such as D_100_, D_90_ for GTV, HR CTV, and IR CTV. Meanwhile, D_0.1cc_, D_1cc_, and D_2cc_ were recommended for evaluating the dose for OAR 1. Recently, Georg et al. 26 reported that some DVH parameters had a good predictive value for the LSE of OAR in MR IGBT. Specifically, D_2cc_ and D_1cc_ can be used to predict all rectal toxicity occurrences, and D_0.1cc_, D_1cc_, and D_2cc_ could only predict severe LSE of the bladder. In our study, we analyzed the relationship between the DVH parameters and the grades of rectal LSE and demonstrated that the D_0.1cc_, D_1cc_, and D_2cc_ values could predict Grade 2 or higher rectal toxicity. Considering that CT‐ and MR‐based contouring of OAR showed no significant difference 21, 23, we propose that D_2cc_ could be more accurate and convenient to predict the occurrence and severity of LSE in 3D BT, which consistent with the data of Lee et al. 10. However, because of some different data in the study of Kim 9, other DVH parameters, such as D_5cc_, D_10cc_, and D_15cc_, also need to be further evaluated in our patients.

With the increased implementation of 3D BT and improved LC rate for locally advanced cervical cancer, some studies have focused on the correlation between the values of DVH parameters and the LSE grade for OARs. Based on CT scans in the treatment of a 2D plan, Kato et al. 27 found that the DVH parameter values were higher in patients with rectal LSE than in those without rectal LSE and that the incidence of complications was also increased in the patients with relatively high DVH values. However, the relationship between dose and severe rectal LSE was not determined. In a study using MR‐based BT, the DVH parameters were demonstrated to be related to the incidence of rectal LSE and severe bladder toxicity 26, and well‐defined dose–response curves were further established for D_2cc_ in the rectum and bladder 28. For CT‐based BT, we reported that the DVH parameters were able to predict Grade 2 and above toxicity of the rectum for the first time. Based on these data, the cutoff value of 73.5 Gy for D_2cc_ might be more appropriate to predict severe LSE.

Radiation proctitis, which is one of the most common complications in cervical cancer radiotherapy, should receive more attention. For traditional BT with a 2D plan, the probability of rectal LSE is high, and a large percentage of patients suffer from Grade 3 and above complications by the ROTG/EORTC scale. The rate even reached 26% in some reports 29. With the increasing application of 3D BT, the clinical outcomes are promising with very low rates of local recurrence and remarkably decreased normal tissue complications 30. Kim et al. compared the LC and late rectal bleeding rates between a CT image‐guided plan and a 2D plan and found that the implementation of a 3D plan not only increased LC, but also decreased rectal complications, especially severe rectal bleeding (2% for 3D vs. 13% for 2D) 6. In our investigation, the D_2cc_ value of the rectum in most patients was limited to <75 Gy, which was in accordance with the recommendation of GYN GEC‐ESTRO, and the mean D_2cc_ of the rectum reached 71 Gy, which was somewhat higher than that used in many other reports. As such, a relatively higher incidence of rectal LSE was shown. In addition, the rate of Grade 3 complications was 10.4%, which seemed to be higher than in other studies using a CT image‐guided plan. These results could partially be explained by the difference between the LENT‐SOMA and RTOG/EORTC staging systems 16, 31. In fact, most patients with Grade 3 rectal complications evaluated by the LENT‐SOMA scale only had light daily bleeding, which could be evaluated as Grade 2 by the RTOG/EORTC standard. In other words, there were few severe rectal complications in our study (RTOG: 2.1%), which also indicated the advantage of a CT image‐guided plan. However, current evaluations of the grade of LSE in many studies are mainly based on the clinical signs and symptoms, such as the severity and frequency of bleeding, which was liable to be influenced by subjective factors; therefore, some objective examinations, such as endoscopy, are needed to further assess the degree of rectal toxicity.

Recently, MR‐based BT showed more ideal outcomes according to all grades of rectal LSE (nearly 10% by LENT‐SOMA) 26. Due to superior soft tissue contrast compared with CT 1, MR‐based BT can ensure sufficient dose targeting for the tumor and a lower dose for the rectum. In our study, as CT can often overestimate the tumor width compared with MR 12, the contours for HR CTV were relatively larger, and thus the rectum received a much higher dose. Considering the actual situation in China, where the number of cervical cancer patients is large and advanced medical resources are not widely utilized, CT‐based BT might be easily applied and generalized. However, considering the satisfactory curative effects and lower rate of rectal LSE, MR‐based BT is strongly recommended in our clinical practice.

In addition, with the development of BT technique, the pattern of BT, such as intracavitary BT, interstitial BT, and combined intracavitary/interstitial BT, the prescribed dose and fractionation schedule, have attracted more and more attention. Some studies showed that combined intracavitary/interstitial BT used in the cases with significant residue after EBRT, which meet patient selection condition in our study, was more conformal with higher dose in target coverage and less critical structures exposure 32, 33. Regarding fractionation schedule, because of some other confounding factors and the complexity of parameters, the toxic effect on rectum was controversial 34, 35. In our data, although there was no difference on rectal toxicity in BT pattern and fractionation schedule, it also needed to be explored in further studies.

There were several limitations in this study. First, this was a small, noncomparative, retrospective study, and the data were based largely on clinical observations. Furthermore, this study was performed in China, and the current findings may not be able to be extrapolated to patients in other parts of the world without further testing. Therefore, well‐designed, long‐term follow‐up, and prospective researches need to be implemented in the future. Finally, further studies need to focus on more parameters to evaluate the dose effect for rectum, and other OARs, such as the bladder and intestine, also need to be involved in.

## Conclusion

In conclusion, CT‐based BT in the treatment of locally advanced cervical cancer shows an excellent pelvic LC rate and favorable toxicity profiles. The DVH parameters can predict the incidence and grades of rectal LSE. D_2cc_ showed an excellent predictive value, and 73.5 Gy for D_2cc_ of the rectum might be considered as an alternative dose limit.

## Conflicts of Interest

The authors have declared no conflicts of interest.
